# Cross-Sectional and Prospective Associations of Carbonated Soft Drink Consumption, *BDNF* Val66Met Polymorphism, and Psychological Distress

**DOI:** 10.3390/nu18162587

**Published:** 2026-08-07

**Authors:** Inkyung Baik

**Affiliations:** 1 Department of Biopharmaceutical Chemistry, College of Science and Technology, Kookmin University, Seoul 02707, Republic of Korea; ibaik@kookmin.ac.kr; Tel.: +82-2-910-4774; 2 Department of Pharmaceutical Engineering, College of Science and Technology, Kookmin University, Seoul 02707, Republic of Korea

**Keywords:** carbonated beverages, tea, coffee, psychological distress, brain-derived neurotrophic factor, prospective studies

## Abstract

**Background/Objectives**: Few prospective studies have investigated the association between carbonated soft drink (CSD) consumption and psychological distress (PD) among adults. The current study aimed to examine this association according to the *BDNF* Val66Met polymorphism and to conduct comparable analyses for coffee and green tea consumption. **Methods**: Cross-sectional data (*n* = 6244) and 6-year prospective data (*n* = 1984) comprising participants aged 43–74 years were analyzed. PD was defined as a Psychosocial Wellbeing Index (PWI) score ≥ 27. Beverage consumption was assessed using a food frequency questionnaire. Modified Poisson regression was employed to estimate multivariable prevalence ratios or risk ratios (RRs) and 95% confidence intervals (CIs) for PD. **Results**: In cross-sectional data, green tea consumption was inversely associated with PD prevalence in the overall study population (*p* for trend < 0.05), and its dose–response relationship was observed exclusively among *BDNF* Met allele (CT and TT) carriers. In 6-year prospective data, CSD consumption was positively associated with PD risk among *BDNF* Met allele carriers only (*p* for trend < 0.01); multivariable RR (95% CI) for PD among *BDNF* Met allele carriers was 1.94 (1.21, 3.10)—(95% CI: 1.09, 3.44) after further applying the Bonferroni correction—for those who consumed ≥ 1 cup/week compared with non-consumers. No prospective association was observed for coffee and green tea consumption in the overall study population and genotype-specific analyses. **Conclusions**: CSD consumption was associated with increased PD risk among individuals carrying the *BDNF* Met allele, which is known to be associated with various stress-related psychological conditions. These findings suggest that CSD consumption may be particularly harmful to individuals with specific genetic factors.

## 1. Introduction

Psychological distress (PD) is a non-specific emotional state characterized by overlapping symptoms of anxiety and depression that adversely affect daily functioning and overall wellbeing [1]. The global prevalence of PD among adults is estimated to exceed 30% and has increased substantially in recent years [2]. A wide range of stressors, including lifestyle factors, may contribute to the development of PD. Several longitudinal studies have demonstrated that a greater accumulation of unhealthy lifestyle behaviors, including insufficient physical activity, poor sleep, unhealthy dietary habits, smoking, and excessive alcohol consumption, is associated with an increased risk of PD development whereas adherence to healthy lifestyle behaviors is associated with a reduced risk [3,4].

Epidemiological studies have consistently suggested that carbonated soft drink (CSD) consumption is associated with adverse mental health outcomes among adolescents [5,6,7,8]. Although data for adults are limited, a cross-sectional study reported positive associations between CSD consumption and both PD and depression [9]. To date, only one longitudinal study has reported the associations of CSD, coffee, and tea consumption with anxiety and depression in adults [10]. The study observed a significant positive association between CSD consumption and anxiety, whereas no significant association was examined for coffee and tea consumption. Therefore, additional prospective studies are needed to clarify these inconclusive findings.

Brain-derived neurotrophic factor (BDNF), a member of the neurotrophin family of growth factors, functions as an important neurotransmitter modulator and plays a critical role in cognitive function [11]. Increasing evidence suggests that BDNF is involved in the pathophysiology of various stress-related mental disorders [12]. An early animal study demonstrated that restraint stress reduced BDNF mRNA expression in the hippocampus [13]. In addition, an epidemiological study reported that questionnaire-based scores of perceived psychological stress were inversely correlated with serum BDNF levels [14], which have been shown to be influenced by a specific polymorphism in the BDNF gene [15]. Among the common *BDNF* polymorphisms, the Val66Met variant has been extensively investigated because of its association with mental health [16]. This missense polymorphism, in which valine at codon 66 is substituted with methionine, impairs BDNF protein secretion [12]. Previous studies have also suggested that coffee, green tea, or their bioactive constituents may influence BDNF expression or secretion [17,18,19,20]. However, whether the *BDNF* Val66Met polymorphism modifies the associations of CSD, coffee, and tea consumption with PD remains unclear because of the limited evidence available.

Therefore, the present study aimed to investigate the associations of CSD, coffee, and green tea consumption with PD through both cross-sectional and prospective study designs. In particular, this study evaluated whether these associations differ according to the *BDNF* Val66Met polymorphism and whether potential interactions between this genetic variant and beverage consumption, as well as between beverage consumption and other risk factors for PD, are significant.

## 2. Participants and Methods

### 2.1. Study Design and Participants

This study utilized data from two population-based prospective cohorts (‘Ansan cohort’ and ‘Ansung cohort’), which are part of the Korean Genome and Epidemiology Study. These ongoing cohort studies are administered by the Korea National Institute of Health (KNIH), and de-identified datasets are available through the KNIH website (https://biobank.nih.go.kr/Desk/. accessed on 1 July 2026) upon official request by researchers. Previous publications have described the participant recruitment procedures and study protocols in detail [21,22]. In brief, male and female residents aged 40 years or older from Ansan and Ansung (Gyeonggi Province, Republic of Korea) were recruited using a two-stage cluster sampling method based on residential district and demographic characteristics. Participants visited designated regional hospitals for baseline interviews and health examinations. Between 2001 and 2002, a total of 10,030 participants completed the baseline assessment and were followed-up biennially. De-identified datasets collected between 2001 and 2020 are currently available for research.

Information on CSD, coffee, and green tea consumption (the study exposures), together with data from the questionnaire used to assess PD (the study outcome), was collected during the follow-up examination between 2005 and 2006. Accordingly, this examination was designated as the baseline for the present study. Initially, 7219 participants provided complete information on the outcome and exposures. After excluding those with missing or invalid data on genotypes (*n* = 878), anthropometric measurements (*n* = 72), or calorie intake (*n* = 25), 6244 participants (3083 in the Ansan cohort and 3161 in the Ansung cohort) were finally included in the cross-sectional study population. Because information on both the outcome and exposures was collected again exclusively in the Ansan cohort during the follow-up examination between 2011 and 2012, this 6-year follow-up assessment was designated as the endpoint for the prospective analysis. Participants with PD at baseline (*n* = 471) were excluded from the prospective data. Among the remaining participants (*n* = 2612), 628 of them were lost to follow-up over the 6-year period, with lack of time reported as the primary reason for non-participation. Consequently, 1984 participants were included in the prospective analysis, corresponding to a follow-up rate of 76% (Supplementary Figure S1 in Supplementary Material).

The original cohort studies were conducted in accordance with the Declaration of Helsinki and relevant local ethical guidelines and regulations governing human research, and their cohort members provided written informed consent before enrollment. The present study was approved by the Institutional Review Board of Kookmin University (KMU-202312-HR-384) with permission from the KNIH.

### 2.2. Psychosocial Wellbeing Index Questionnaire for Assessing Psychological Distress

PD, the primary outcome of this study, was assessed based on the Psychosocial Wellbeing Index (PWI) questionnaire, which has been developed based on the General Health Questionnaire (GHQ) and validated in previous studies exhibiting a high correlation between PWI and GHQ scores [23,24]. The questionnaire is composed of 18 items assessing psychological states that participants have recently experienced or felt over the past few weeks, including mental wellbeing, anxiety, worry, irritability, depressed mood, sleep quality, fatigue, energy level, self-confidence, feeling happiness, positive thinking, life satisfaction, willingness to perform tasks, and problem-solving ability. Each item is scored on a four-point scale ranging from 0 to 3 (always, mostly, somewhat, or never) with a higher score indicating a higher level of PD. The total PWI score ranges from 0 to 54. Consistent with previous studies [23,25], participants with a PWI score of 27 or higher were classified as having PD.

### 2.3. Assessment of Carbonated Soft Drink, Coffee, and Green Tea Consumption

The primary exposures of interest were CSD, coffee, and green tea consumption. During the baseline examination conducted between 2005 and 2006, dietary intake was assessed using a food frequency questionnaire (FFQ) developed and validated by the Korea Disease Control and Prevention Agency (Seoul, Korea) [21]. The FFQ asks participants to recall the average frequency (“almost never,” “once a month,” “2–3 times a month,” “1–2 times a week,” “3–4 times a week,” “5–6 times a week,” “once a day,” “twice a day,” or “3 times a day”) and amount of foods and dishes (“larger than,” “equal to,” or “smaller than” the standard serving size) consumed over the past year. The standard serving size was defined as 1 cup for coffee and green tea and 200 mL for CSDs, including coke and soda. Based on the consumption distribution of each beverage, daily or weekly consumption amounts were calculated and classified as follows; no consumption, <1 cup/week, and ≥1 cup/week for CSDs; no consumption, <1 cup/day, 1–1.9 cups/day, and ≥2 cups/day for coffee; no consumption, <4 cups/week, 4–6 cups/week, and ≥7 cups/week for green tea. Baseline consumption data were used in the cross-sectional and prospective analyses. Additionally, the average consumption amounts were calculated using data from both the baseline and follow-up assessments in the prospective analysis. In the follow-up assessment, beverage consumption was assessed using a caffeine-focused FFQ, which collected detailed information on the frequency and quantity of 18 beverage types consumed [26]. While the standard serving size for beverages were identical between the baseline FFQ and the caffeine-focused FFQ, the caffeine-focused FFQ included a greater number of beverage items, including CSDs, coffee, and green tea. In this study, daily or weekly consumption was calculated using data exclusively on the same types of beverages from the two questionnaires.

### 2.4. BDNF Gene Val66Met Genotyping

Details regarding the preparation of DNA samples, genotyping procedures, and quality control methods to generate single nucleotide polymorphisms (SNPs) for the Ansan and Ansung cohorts have been described previously [27]. In brief, DNA extracted from whole blood samples provided by 9993 participants was genotyped using Genome Wide Human SNP Array 5.0 (Affymetrix, Inc., Santa Clara, CA, USA). Following quality control procedures, including a genotype call rate of at least 96%, verification of sex concordance, and an average pairwise identity-by-state value ≤ 0.8, genomic data for 8842 individuals comprising 500,568 SNPs were retained for analysis. Genotype information for the *BDNF* Val66Met polymorphism (rs6265: C > T), which conformed to the Hardy–Weinberg equilibrium (*p*-value = 0.46), was extracted from the original SNP data.

### 2.5. Confounding Variables

Potential confounding variables included demographic and socioeconomic characteristics (age, sex, marital status, monthly income, education level, and occupation), lifestyle factors (smoking status, alcohol consumption, and physical activity), prevalent chronic diseases, body mass index (BMI), and calorie intake. These data were collected through standardized questionnaire-based interviews and health examinations conducted by trained personnel. Detailed methods for assessing alcohol consumption have been reported previously [22]. Average daily calorie intake was estimated using FFQ data with the food composition database published by the Rural Development Administration of Korea [28]. Information regarding disease history and diagnosis, treatment method, and medication for diabetes mellitus (DM), hypertension (HTN), cardiovascular disease (CVD), and cancer was obtained using standardized questionnaires. The presence of DM and HTN was additionally assessed using serum glucose measurements obtained with the Cobas analyzer (Roche Diagnostics, Manheim, Germany) and blood pressure measurements performed during the health examination. BMI was calculated as body weight (kg) divided by height squared (m^2^).

### 2.6. Statistical Analysis

Baseline characteristics were summarized according to PWI scores and are presented as the mean ± standard deviation for continuous variables or percentage for categorical variables. Differences among the three groups were evaluated using the chi-square test and analysis of variance (ANOVA) for categorical and continuous variables, respectively. Linear trends were assessed using the Cochran–Armitage and ANOVA trend tests.

Consistent with previous findings [12], the Met allele (CT and TT) was reported to be associated with reduced BDNF secretion. The frequencies of the *BDNF* Val66Met genotype were 0.3 for CC, 0.5 for CT, and 0.2 for TT; however, the CT and TT genotype groups were combined due to a small number of cases in prospective analysis.

Associations between beverage consumption and PD were evaluated using modified Poisson regression to estimate multivariable prevalence ratios (PRs) or risk ratios (RRs) and 95% confidence intervals (CIs). Multivariable models were adjusted for age (continuous), sex, marital status (married and living with a spouse or other), monthly income (≤2 × 10^6^ or >2 × 10^6^ won based on the eligibility threshold for livelihood assistance for a family of four; https://www.mohw.go.kr. accessed on 1 July 2026), educational level (≤middle school or ≥high school), occupational type (office worker or other), BMI (continuous), smoking (pack-years, continuous), alcohol consumption (five categories: non-drinker, former drinker or non-responder, or current drinker consuming ≤15 g/day, 15.1–30 g/day, or >30 g/day), daily physical activity (four categories: none, <30 min/day, 30–59 min/day, or ≥60 min/day), daily calorie intake (quintiles), prevalent DM and HTN or physician-diagnosed CVD and cancer in the past 2 years (yes or no), and *BDNF* Val66Met genotype (CC vs. CT and TT). Interactions between beverage consumption, confounding variables, and *BDNF* Val66Met polymorphism were further evaluated by including an interaction term (1 degree of freedom) in the model. In addition, Bonferroni correction was applied to adjust the significance level for post-hoc tests, thereby preventing false positives (Type I errors) that could arise during multiple statistical comparisons. All statistical analyses were conducted using SAS 9.4 (SAS Institute, Cary, NC, USA). Statistical significance was defined as a two-sided *p*-value < 0.05.

## 3. Results

### 3.1. Baseline Characteristics According to Psychosocial Wellbeing Index

Among the 6244 study participants (3235 females and 3009 males), 1097 cases of PD (320 cases in CC carriers, 564 cases in CT carriers, and 213 cases in TT carriers) were identified at baseline. The baseline characteristics of the three groups of PWI scores (<9, 9–26, and ≥27) are summarized in Table 1. Participants with higher PWI scores generally had lower BMI, were unmarried or not living with a spouse, and were less likely to smoke. They also tended to have lower levels of monthly income and educational attainment and were more likely to be employed in non-office occupations. In addition, participants with higher PWI scores were more likely to abstain from alcohol, engage in less physical activity, consume less calories, and consume lower amounts of coffee and green tea.

### 3.2. Cross-Sectional Associations Between Beverage Consumption and Psychological Distress

Table 2 presents the cross-sectional associations between CSD, coffee, and green tea consumption and PD prevalence in the overall study population and according to the *BDNF* Val66Met genotype. In multivariable models for the overall study population, green tea consumption was inversely associated with PD prevalence (*p* for trend < 0.05). In comparison with non-consumers of green tea, multivariable PRs (95% CIs) were 0.75 (0.61, 0.92)–(95% CI: 0.57, 0.99) after further applying the Bonferroni correction—for 4–6 cups/week and 0.80 (0.67, 0.94)–(95% CI: 0.64, 0.99) after further applying the Bonferroni correction—for ≥7 cups/week. This inverse dose–response relationship between green tea consumption and PD prevalence was particularly evident among carriers of the *BDNF* Met allele (CT and TT) (*p* for trend < 0.05); multivariable PR (95% CI) was 0.77 (0.63, 0.94)–(95% CI: 0.59, 1.01) after further applying the Bonferroni correction—for ≥7 cups/week. In contrast, no significant linear trend was observed in multivariable analysis for CSD consumption; the multivariable PRs (95% CIs) for PD prevalence were 1.16 (1.01, 1.33)–(95% CI: 0.98, 1.37) after further applying the Bonferroni correction—for < 1 cup/week and 1.16 (0.97, 1.39) for ≥1 cup/week in the overall study population. Genotype-specific analysis further demonstrated a positive association between CSD consumption and PD prevalence exclusively among *BDNF* Met allele (CT and TT) carriers; multivariable PR (95% CI) was 1.22 (1.04, 1.42)–(95% CI: 1.01, 1.47) after further applying the Bonferroni correction—for < 1 cup/week compared with non-consumers of CSD. However, no significant association between coffee consumption and PD prevalence was observed after multivariable adjustment.

### 3.3. Prospective Associations Between Beverage Consumption and Psychological Distress Risk

Table 3 summarizes the prospective associations between baseline consumption of CSDs, coffee, and green tea and the 6-year risk of PD among the 1984 participants included in the prospective analysis, together with the genotype-stratified results. Among these participants (924 females and 1060 males), 207 incident cases of PD (69 cases in CC carriers, 97 cases in CT carriers, and 41 cases in TT carriers) were identified in the follow-up examination. In the multivariable analysis of all participants, a linear trend was observed only for the association between CSD consumption and PD risk (*p* for trend = 0.08). In particular, this association was evident among *BDNF* Met allele (CT and TT) carriers showing a dose–response relationship (*p* for trend < 0.01). CSD consumption ≥ 1 cup/week significantly increased the risk of PD among *BDNF* Met allele (CT and TT) carriers; the multivariable RR (95% CI) for PD risk was 1.94 (1.21, 3.10)–(95% CI: 1.09, 3.44) after further applying the Bonferroni correction—for participants who consumed ≥ 1 cup/week compared with non-consumers. After further adjusting for baseline PWI scores, the multivariable RR (95% CI) for PD risk was 1.89 (1.19, 2.99)–(95% CI: 1.08, 3.31) after further applying the Bonferroni correction—for participants who consumed ≥ 1 cup/week compared with non-consumers among *BDNF* Met allele (CT and TT) carriers (data are available on request). However, coffee and green tea consumption was not associated with PD risk in the multivariable analysis for all participants or the genotype-specific analysis. Additional results regarding the average consumption are available in Supplementary Material (Supplementary Table S1).

**Figure 1:**
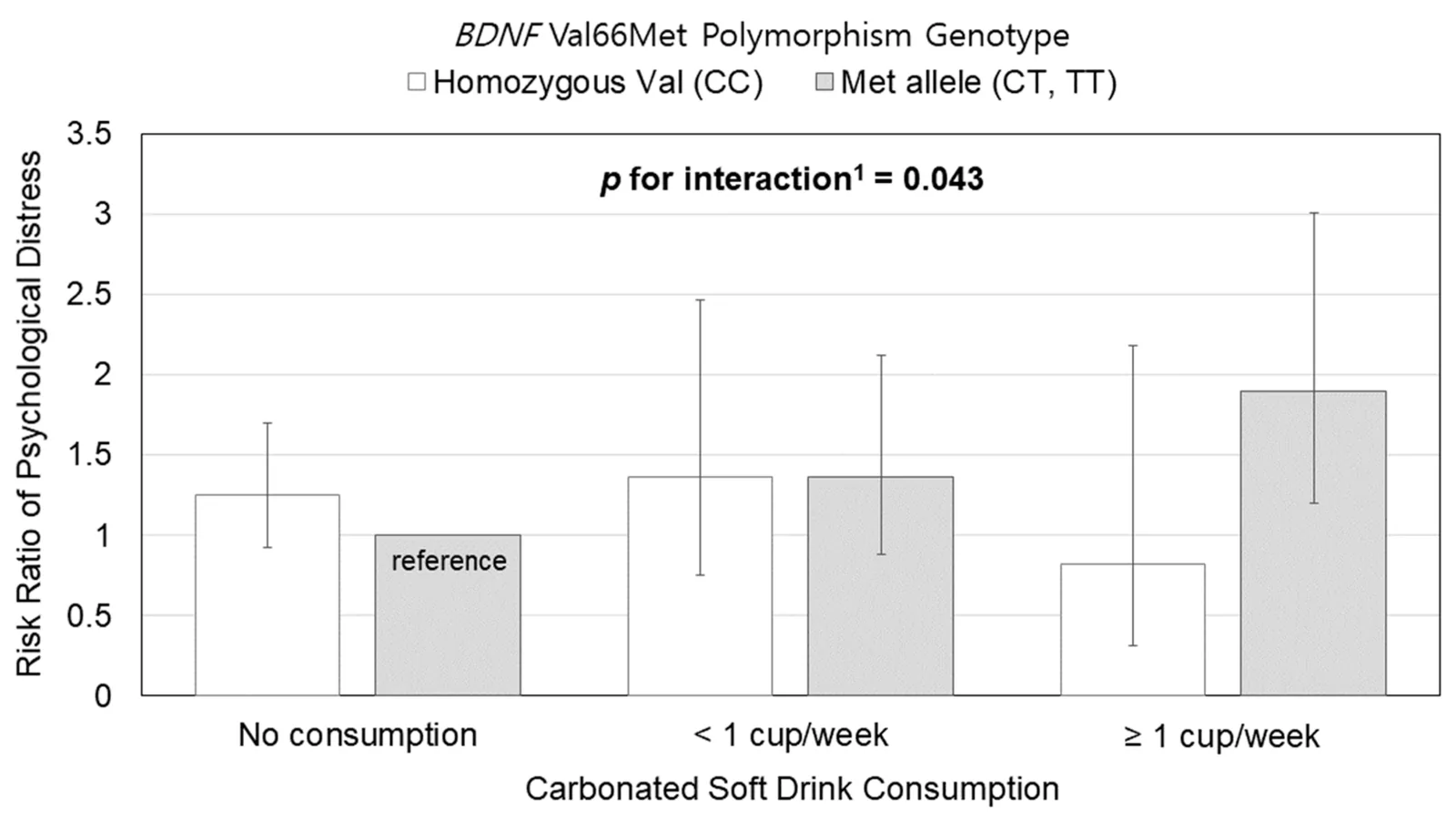
Prospective association between carbonated soft drink consumption and the risk of psychological distress (Psychosocial Wellbeing Index ≥ 27) according to *BDNF* Val66Met genotype (CC vs. CT and TT) among 1984 participants. Risk ratios and 95% confidence intervals were estimated after adjustment for age (continuous), sex, marital status (married and living with a spouse or other), monthly income (≤2 × 10^6^ or >2 × 10^6^ won), educational level (≤middle school or ≥high school), occupational type (office worker or other), body mass index (continuous), smoking (pack-years, continuous), alcohol consumption (five categories: non-drinker, former drinker or non-responder, or current drinker: consuming ≤ 15 g/day, 15.1–30 g/day, or >30 g/day), daily physical activity (four categories: none, <30 min/day, 30–59 min/day, or ≥60 min/day), daily calorie intake (quintiles), prevalent diabetes mellitus and hypertension or physician-diagnosed cardiovascular disease and cancer in the past 2 years (yes or no), *BDNF* Val66Met genotype (CC vs. CT and TT), and coffee and green tea consumption. The reference group comprised participants with the Met allele (CT and TT) who did not consume carbonated soft drinks. ^1^ *p*-value for the interaction between carbonated soft drink consumption and *BDNF* Val66Met genotype.

The results of further analysis stratified by the *BDNF* Val66Met genotypes are presented in Figure 1. Multivariable RRs for PD risk were estimated using participants with the *BDNF* Met allele (CT and TT) who did not consume CSDs as the reference. The RRs (95% CIs) were 1.25 (0.92, 1.70) for no consumption, 1.36 (0.75, 2.46) for <1 cup/week, and 0.82 (0.31, 2.18) for ≥1 cup/week among homozygous Val (CC) carriers. Among *BDNF* Met allele (CT and TT) carriers, the corresponding RRs (95% CIs) were 1.36 (0.88, 2.12) for <1 cup/week and 1.90 (1.20, 3.01) for ≥1 cup/week. A statistically significant interaction (*p* = 0.043) between CSD consumption and the *BDNF* Val66Met genotype was identified (Figure 1). In an additional analysis further adjusted for baseline PWI scores, the significance of the interaction term was more pronounced (*p* = 0.019) (data are available on request).

### 3.4. Joint Associations of Carbonated Soft Drink Consumption and Confounding Factors with Psychological Distress Risk

Table 4 presents the exploratory analysis results of interactions between CSD consumption and selected confounding factors, including age, sex, marital status, monthly income, educational level, occupational type, BMI, smoking, alcohol consumption, physical activity, and diagnosis of chronic diseases in relation to PD risk. No significant interaction was observed between CSD consumption and any of the evaluated confounding variables. Nevertheless, significant positive associations between CSD consumption and PD risk were identified among female participants, participants with lower BMI, smokers, and never-drinkers of alcoholic beverages compared with their respective reference groups. Regardless of CSD consumption, participants with lower income, lower educational level, or chronic diseases had significantly higher risks of developing PD.

## 4. Discussion

The present study used data from population-based cohorts to investigate the cross-sectional and prospective associations of CSD, coffee, and green tea consumption among adults with PD according to the *BDNF* Val66Met polymorphism. The association between CSD consumption and PD was more pronounced in the 6-year prospective analysis than in the cross-sectional analysis and was evident among *BDNF* Met allele (CT and TT) carriers but not among homozygous Val (CC) carriers. In particular, compared with non-consumers, *BDNF* Met allele (CT and TT) carriers who consumed ≥ 1 cup/week of CSDs exhibited a 1.9-fold higher risk of developing PD. In contrast, although green tea consumption was inversely associated with PD in the cross-sectional analysis, no significant prospective association was observed. Notably, the inverse dose–response relationship between green tea consumption and PD prevalence was more evident among *BDNF* Met allele (CT and TT) carriers than among homozygous Val (CC) carriers.

PD is considered to result from the complex interplay of multiple stressors and is characterized by emotional, cognitive, and physical symptoms arising from excessive or prolonged stress [1]. A previous cross-sectional study using surveillance data from Australian adults reported a 1.7-fold higher odds of PD prevalence was associated with consumption of ≥500 mL/day of CSDs compared with no consumption [9]. Furthermore, a 1-year longitudinal study of a European adult population found that consumption of 200 mL/day of sugar-sweetened CSDs was associated with a 2.5-fold higher odds of anxiety, whereas coffee and tea consumption was not associated with anxiety [10].

In the present prospective analysis, no significant association was observed between CSD, coffee, or green tea consumption and PD risk in the overall study population. However, genotype-specific analyses revealed a significant dose–response relationship between CSD consumption and PD risk among individuals with *BDNF* Met allele. Specifically, individuals carrying this allele who consumed CSDs ≥ 1 cup (200 mL)/week, which represents a relatively low intake compared with the consumption observed in previous studies [9,10], had more PD events than non-consumers. According to a previous study analyzing data from Korean national health surveys, the average CSD consumption was reported to be 12–17 mL/day in Korean adults aged 50–64 years [29]. Among the study participants included in the prospective analysis, approximately 76% were non-consumers of CSDs and 11% were consumers of ≥ 1 cup/week. This low level of CSD consumption might not be sufficient to significantly increase PD risk in the overall study population, but it could elevate the risk significantly among individuals with a specific genetic polymorphism. In addition, the association between CSD consumption and PD risk was significant among female participants, participants with lower BMI, smokers, and never-drinkers of alcoholic beverages while lower income, lower educational attainment, and the presence of chronic diseases were associated with PD risk regardless of CSD consumption. However, unlike these confounding factors, the *BDNF* Val66Met polymorphism emerged as a significant effect modifier in the association between CSD consumption and PD risk. In the cross-sectional analysis, green tea consumption of ≥ 4 cups/week was inversely associated with the prevalence of PD in the overall study population while no corresponding association was observed with the incidence of PD in the prospective analysis, partly due to limited statistical power resulting from the small number of cases. Another possible explanation for these discrepant findings is reverse causation. For example, individuals experiencing PD at baseline may have increased their green tea consumption in an attempt to relieve stress. Because participants with PD at baseline were excluded from the prospective analysis, such behavioral changes would not have influenced the results of prospective analysis.

Accumulating evidence suggests that CSD consumption adversely affects mental health in adolescents and is associated with PD, anxiety, hyperactivity, behavioral problems, and suicide attempts [5,6,7,8]. However, evidence in adults remains limited, particularly regarding the potential modifying role of genetic factors. Several biological mechanisms may explain the observed association between CSD consumption and PD risk, as well as the modifying effects of the *BDNF* Val66Met polymorphism. Sugar-containing CSDs may activate the hypothalamic–pituitary–adrenal axis, resulting in increased cortisol secretion and subsequent reductions in BDNF expression [30,31]. According to a review article, the *BDNF* Met allele impairs the ability to maintain optimal levels of BDNF expression—which are essential for neuroplasticity and mood regulation—by inhibiting activity-dependent BDNF secretion [12]. Based on these propositions, individuals with this specific genetic factor may be particularly vulnerable to the effects of CSD consumption. However, as the proposed mechanism remains at the hypothesis stage, further research is needed to elucidate the biological mechanisms underlying the association between CSD consumption, the *BDNF* Val66Met polymorphism, and PD risk with the measurement of stress-related biomarkers and circulating BDNF levels. Conversely, a 6-week intervention study involving adolescents with stuttering reported that consumption of 6 cups/day of green tea reduced adrenal stress hormones, including cortisol, and improved psychological well-being [32]. These findings indicate that green tea consumption may alleviate stress by modulating stress hormone responses. Catechins (particularly epigallocatechin-3-gallate) and L-theanine have been suggested as key components of green tea contributing to the neuroprotective effects and the alleviation of symptoms associated with depression, stress, and anxiety; however, their effects on serum BDNF levels remain unclear due to limited data [33]. Furthermore, the influence of *BDNF* polymorphisms on the neuroprotective effects of green tea or its bioactive components has not yet been reported.

This study has several strengths. It employed a prospective study design, incorporated a functional genetic polymorphism into the analyses, and adjusted for a comprehensive range of potential confounding variables. Nevertheless, several limitations should be acknowledged. First, PD was defined using the PWI questionnaire, which, although developed on the basis of the GHQ widely used to assess PD [23,24], differs from other commonly used instruments. Several previous studies have used the Kessler Psychological Distress Scale to define PD [4,9]. Consequently, direct comparisons with previous studies should be made with caution. Second, the FFQ used in this study did not distinguish between sugar-sweetened beverages and artificially sweetened beverages. However, given that artificially sweetened beverages were first introduced in Korea in April 2006, it is assumed that the vast majority of CSD consumption at baseline consisted of sugar-sweetened beverages. Third, there might be residual confounders, such as history of psychiatric disorders or sleep disorders and other dietary factors; sugar-sweetened beverages might be part of an overall unhealthy dietary pattern. Another limitation is the 24% loss-to-follow-up rate and the use of data from a single cohort in the prospective analysis. Because the study population consisted exclusively of middle-aged or older Korean adults, there may be limitations in generalizing the findings of this study to groups with different age ranges, racial or cultural backgrounds, or residential locations.

## 5. Conclusions

In summary, this study demonstrated a prospective dose–response relationship between CSD consumption and an increased risk of developing PD, as well as a cross-sectional dose–response association between green tea consumption and reduced PD prevalence. These associations were particularly evident among individuals with *BDNF* Met allele. The findings suggest that individuals with specific genetic factors may be more vulnerable to the stress-related mental condition associated with CSD consumption. Future prospective studies are needed to understand the potential effects of the habitual consumption of various beverages, including CSDs and green tea, and to further elucidate the underlying biological mechanisms involving the role of *BDNF* polymorphisms.

## Figures and Tables

**Table 1:** Baseline characteristics of 6244 study participants according to Psychosocial Wellbeing Index.

Characteristics	Categories of Psychosocial Wellbeing Index [Median]			*p* for Trend ^1^
	<9 [5]	9–26 [17]	≥27 [31]	
Number of participants (% of total)	942 (15.1)	4205 (67.3)	1097 (17.6)	
Age, years	57.5 ± 8.8	55.4 ± 8.7	57.5 ± 9.1	0.95
Female, %	41.8	50.7	64.7	<0.001
Body mass index, kg/m^2^	24.9 ± 3.0	24.5 ± 3.0	24.2 ± 3.3	<0.001
Married and living with a spouse, %	89.7	90.1	82.5	<0.001
Low income ^2^, %	58.3	53.3	67.3	<0.001
High school education or higher, %	43.3	46.8	30.7	<0.001
Office occupation, %	15.6	19.1	10.5	<0.001
Current smoker, %	18.7	18.4	19.8	0.49
Lifetime smoking exposure, pack-years	11.7 ± 18.9	10.3 ± 17.7	9.2 ± 16.8	<0.01
Current alcohol drinker, %	48.5	49.3	42.1	<0.01
Moderate physical activity, %	43.0	38.1	26.1	<0.001
Underlying medical condition ^3^, %	33.2	31.9	32.9	0.92
Calorie intake, kcal/day	1867.4 ± 579.3	1794.8 ± 539.6	1645.5 ± 524.6	<0.001
*BDNF* Met allele (CT, TT) genotype, %	68.6	70.3	70.8	0.28
Consumers of beverages, %				
Carbonated soft drinks	28.7	30.3	31.5	0.16
Coffee	82.5	81.5	75.2	<0.001
Green tea	66.4	69.7	59.5	<0.001

Values are presented as mean ± standard deviation or %. ^1^ *p*-values were obtained using the Cochran–Armitage trend test for categorical variables and analysis of variance trend test for continuous variables. ^2^ Average monthly income < 2 × 10^6^ won. ^3^ Prevalent diabetes mellitus and hypertension or physician-diagnosed cardiovascular disease and cancer in the past 2 years.

**Table 2:** Cross-sectional associations between beverage consumption and psychological distress (Psychosocial Wellbeing Index ≥ 27) for 6244 participants, overall and stratified by *BDNF* Val66Met genotype.

Beverages	Cases/Non-Cases ^1^	PR (95% CI) for All		PR (95% CI) Stratified by Genotype	
	[Cases/Non-Cases] ^2^	Model 1	Model 2	Model 2 for CC	Model 2 for CT/TT
Carbonated soft drinks					
No consumption	751/3602 [226/1078:525/2524]	Reference	Reference	Reference	Reference
<1 cup/week	221/919 [56/277:165/642]	1.18 (1.03, 1.35)	1.16 (1.01, 1.33)	1.04 (0.79, 1.36)	1.22 (1.04, 1.42)
≥1 cup/week	125/626 [38/192:87/434]	1.11 (0.93, 1.32)	1.16 (0.97, 1.39)	1.16 (0.84, 1.61)	1.16 (0.93, 1.44)
*p* for trend		0.25	0.10	0.37	0.17
Coffee					
No consumption	272/944 [83/298:189/646]	Reference	Reference	Reference	Reference
<1 cup/day	247/1181 [69/366:178/815]	0.85 (0.73, 0.99)	0.86 (0.74, 1.01)	0.84 (0.63, 1.13)	0.87 (0.73, 1.04)
1–1.9 cups/day	372/1926 [112/554:260/1372]	0.81 (0.70, 0.93)	0.87 (0.76, 1.00)	0.92 (0.70, 1.19)	0.86 (0.73, 1.02)
≥2 cups/day	206/1096 [56/329:150/767]	0.92 (0.78, 1.09)	0.98 (0.83, 1.17)	0.91 (0.66, 1.27)	1.03 (0.84, 1.27)
*p* for trend		0.64	0.68	0.92	0.45
Green tea					
No consumption	444/1593 [137/476:307/1117]	Reference	Reference	Reference	Reference
<4 cups/week	356/1599 [91/465:265/1134]	0.85 (0.75, 0.96)	0.92 (0.81, 1.04)	0.86 (0.67, 1.10)	0.94 (0.81, 1.09)
4–6 cups/week	101/649 [27/208:74/441]	0.65 (0.53, 0.80)	0.75 (0.61, 0.92)	0.63 (0.42, 0.93)	0.83 (0.65, 1.05)
≥7 cups/week	196/1306 [65/398:131/908]	0.64 (0.55, 0.75)	0.80 (0.67, 0.94)	0.85 (0.63, 1.14)	0.77 (0.63, 0.94)
*p* for trend		<0.001	<0.05	0.41	<0.05

Abbreviations: PR, prevalence ratio; CI, confidence interval. ^1^ Number of PD cases and non-cases in the overall study population; ^2^ Number of PD cases and non-cases among CC carriers vs. (indicated as ‘:’) CT/TT carriers. Model 1 was adjusted for age (continuous) and sex. Model 2 was adjusted for age (continuous), sex, marital status (married and living with a spouse or other), monthly income (≤2 × 10^6^ or >2 × 10^6^ won), educational level (≤middle school or ≥high school), occupational type (office worker or other), body mass index (continuous), smoking (pack-years, continuous), alcohol consumption (five categories: non-drinker, former drinker or non-responder, or current drinker: consuming ≤ 15 g/day, 15.1–30 g/day, or >30 g/day), daily physical activity (four categories: none, <30 min/day, 30–59 min/day, or ≥60 min/day), daily calorie intake (quintiles), prevalent diabetes mellitus and hypertension or physician-diagnosed cardiovascular disease and cancer in the past 2 years (yes or no), *BDNF* Val66Met genotype (CC vs. CT and TT), and consumption of carbonated soft drinks, coffee, and green tea. The reference group is non-consumers for each beverage in overall analysis and genotype-specific analysis.

**Table 3:** Prospective associations between baseline beverage consumption and the risk of psychological distress (Psychosocial Wellbeing Index ≥ 27) for 1984 participants, overall and stratified by *BDNF* Val66Met genotype.

Beverages	Cases/Non-Cases ^1^	RR (95% CI) for All		RR (95% CI) Stratified by Genotype	
	[Cases/Non-Cases] ^2^	Model 1	Model 2	Model 2 for CC	Model 2 for CT/TT
Carbonated soft drinks					
No consumption	151/1347 [54/407:97/940]	Reference	reference	reference	reference
<1 cup/week	32/240 [11/82:21/158]	1.25 (0.87, 1.79)	1.26 (0.88, 1.81)	1.14 (0.61, 2.13)	1.37 (0.88, 2.13)
≥1 cup/week	24/190 [4/62:20/128]	1.28 (0.85, 1.94)	1.45 (0.95, 2.22)	0.77 (0.28, 2.13)	1.94 (1.21, 3.10)
*p* for trend		0.23	0.08	0.63	<0.01
Coffee					
No consumption	29/273 [13/89:16/184]	Reference	reference	reference	reference
<1 cup/day	33/315 [9/113:24/202]	0.99 (0.62, 1.59)	0.96 (0.60, 1.55)	0.58 (0.25, 1.31)	1.33 (0.73, 2.42)
1–1.9 cups/day	99/755 [31/226:68/529]	1.27 (0.85, 1.88)	1.26 (0.83, 1.91)	0.82 (0.42, 1.59)	1.57 (0.91, 2.72)
≥2 cups/day	46/434 [16/123:30/311]	1.19 (0.75, 1.88)	1.12 (0.67, 1.86)	0.89 (0.40, 1.98)	1.26 (0.65, 2.46)
*p* for trend		0.32	0.52	0.72	0.71
Green tea					
No consumption	56/365 [21/118:35/247]	Reference	reference	reference	reference
<4 cups/week	60/486 [15/144:45/342]	0.82 (0.58, 1.16)	0.86 (0.61, 1.21)	0.65 (0.35, 1.21)	0.93 (0.61, 1.42)
4–6 cups/week	28/268 [8/89:20/179]	0.72 (0.47, 1.11)	0.80 (0.51, 1.24)	0.54 (0.24, 1.20)	0.90 (0.53, 1.54)
≥7 cups/week	63/658 [25/200:38/458]	0.69 (0.49, 0.97)	0.78 (0.55, 1.10)	0.86 (0.49, 1.51)	0.70 (0.45, 1.09)
*p* for trend		0.07	0.25	0.93	0.09

Abbreviations: RR, risk ratio; CI, confidence interval. ^1^ Number of PD cases and non-cases in the overall study population; ^2^ Number of PD cases and non-cases among CC carriers vs. (indicated as ‘:’) CT/TT carriers. Model 1 was adjusted for age (continuous) and sex. Model 2 was adjusted for age (continuous), sex, marital status (married and living with a spouse or other), monthly income (≤2 × 10^6^ or >2 × 10^6^ won), educational level (≤middle school or ≥high school), occupational type (office worker or other), body mass index (continuous), smoking (pack-years, continuous), alcohol consumption (five categories: non-drinker, former drinker or non-responder, or current drinker: consuming ≤ 15 g/day, 15.1–30 g/day, or >30 g/day), daily physical activity (four categories: none, <30 min/day, 30–59 min/day, or ≥60 min/day), daily calorie intake (quintiles), prevalent diabetes mellitus and hypertension or physician-diagnosed cardiovascular disease and cancer in the past 2 years (yes or no), *BDNF* Val66Met genotype (CC vs. CT and TT), and consumption of carbonated soft drinks, coffee, and green tea. The reference group is non-consumers for each beverage in overall analysis and genotype-specific analysis.

**Table 4:** Exploratory analysis for the joint associations of carbonated soft drink consumption and potential confounding factors with the risk of psychological distress (Psychosocial Wellbeing Index ≥ 27) among 1984 participants.

Confounding Factors	Categories	Carbonated Soft Drinks		*p*
		No Consumption	Consumption	for Interaction
Age	<60 years	Reference	1.38 (0.99, 1.93)	0.70
	≥60 years	1.04 (0.71, 1.53)	1.25 (0.68, 2.30)	
Sex	Male	Reference	1.26 (0.83, 1.90)	0.67
	Female	1.43 (0.91, 2.25)	2.03 (1.20, 3.46)	
Marital status	Married ^1^	Reference	1.24 (0.90, 1.70)	0.11
	Other	0.74 (0.42, 1.28)	1.85 (0.94, 3.61)	
Low income	No	Reference	1.19 (0.81, 1.76)	0.33
	Yes	1.53 (1.09, 2.15)	2.43 (1.53, 3.84)	
Educational level	High	Reference	1.39 (0.92, 2.09)	0.77
	Low	1.44 (1.02, 2.02)	1.83 (1.19, 2.82)	
Occupational type	Office worker	Reference	0.96 (0.52, 1.76)	0.20
	Non-office worker	0.95 (0.63, 1.41)	1.42 (0.91, 2.21)	
Body mass index	<25 kg/m^2^	Reference	1.51 (1.02, 2.22)	0.36
	≥25 kg/m^2^	1.02 (0.75, 1.38)	1.17 (0.75, 1.81)	
Smoker	Never	Reference	1.40 (0.95, 2.05)	0.65
	Ever	1.50 (0.92, 2.44)	1.83 (1.04, 3.23)	
Alcohol drinker	Never	Reference	1.54 (1.00, 2.38)	0.39
	Ever	1.27 (0.90, 1.80)	1.52 (0.97, 2.39)	
Physical activity	No	Reference	1.28 (0.87, 1.89)	0.79
	Yes	0.84 (0.62, 1.14)	1.16 (0.75, 1.81)	
Chronic diseases ^2^	No	Reference	1.22 (0.82, 1.82)	0.50
	Yes	1.39 (1.01, 1.89)	2.07 (1.36, 3.17)	

Data are presented as multivariable-adjusted risk ratios with 95% confidence intervals. Risk ratios and 95% confidence intervals were estimated after adjustment for age (continuous), sex, marital status (married and living with a spouse or other), monthly income (≤2 × 10^6^ or >2 × 10^6^ won), educational level (≤middle school or ≥high school), occupational type (office worker or other), body mass index (continuous), smoking (pack-years, continuous), alcohol consumption (five categories: non-drinker, former drinker or non-responder, or current drinker: consuming ≤ 15 g/day, 15.1–30 g/day, or >30 g/day), daily physical activity (four categories: none, <30 min/day, 30–59 min/day, or ≥60 min/day), daily calorie intake (quintiles), prevalent diabetes mellitus and hypertension or physician-diagnosed cardiovascular disease and cancer in the past 2 years (yes or no), and coffee and green tea consumption. ^1^ Married and living with a spouse. ^2^ Prevalent diabetes mellitus and hypertension or physician-diagnosed cardiovascular disease and cancer in the past 2 years.

## Data Availability

Raw data are available through the National Biobank of Korea, following the data distribution procedure described on its website (https://biobank.nih.go.kr/eng, accessed on 27 March 2026).

## Supplementary Materials

The following supporting information can be downloaded at: https://www.mdpi.com/article/10.3390/nu18162587/s1, Figure S1. Flow diagram for study participants; Table S1. Prospective associations between average consumption of the baseline and follow-up assessments and the risk of psychological distress (Psychosocial Wellbeing Index ≥ 27) for 1984 participants, overall and stratified by BDNF Val66Met genotype.

## Abbreviations

The following abbreviations are used in this manuscript:

ANOVA	analysis of variance
BDNF	brain-derived neurotrophic factor
BMI	body mass index
CI	confidence interval
CSD	carbonated soft drink
CVD	cardiovascular disease
DM	diabetes mellitus
FFQ	food frequency questionnaire
GHQ	General Health Questionnaire
HTN	hypertension
KNIH	Korea National Institute of Health
KoGES	Korean Genome and Epidemiology Study
PD	psychological distress
PR	prevalence ratio
PWI	psychosocial Wellbeing Index
RR	risk ratio
SNP	single nucleotide polymorphism
