# Supplementary material for: Cross-Sectional and Prospective Associations of Carbonated Soft Drink Consumption, BDNF Val66Met Polymorphism, and Psychological Distress
Source: Nutrients. 2026 Aug 7;18(16):2587. doi: 10.3390/nu18162587 (PMC13516585; doi:10.3390/nu18162587)
Supplement: Supplementary file 1 [file nutrients-18-02587-s001.zip › nutrients-4453052-supplementary.pdf]

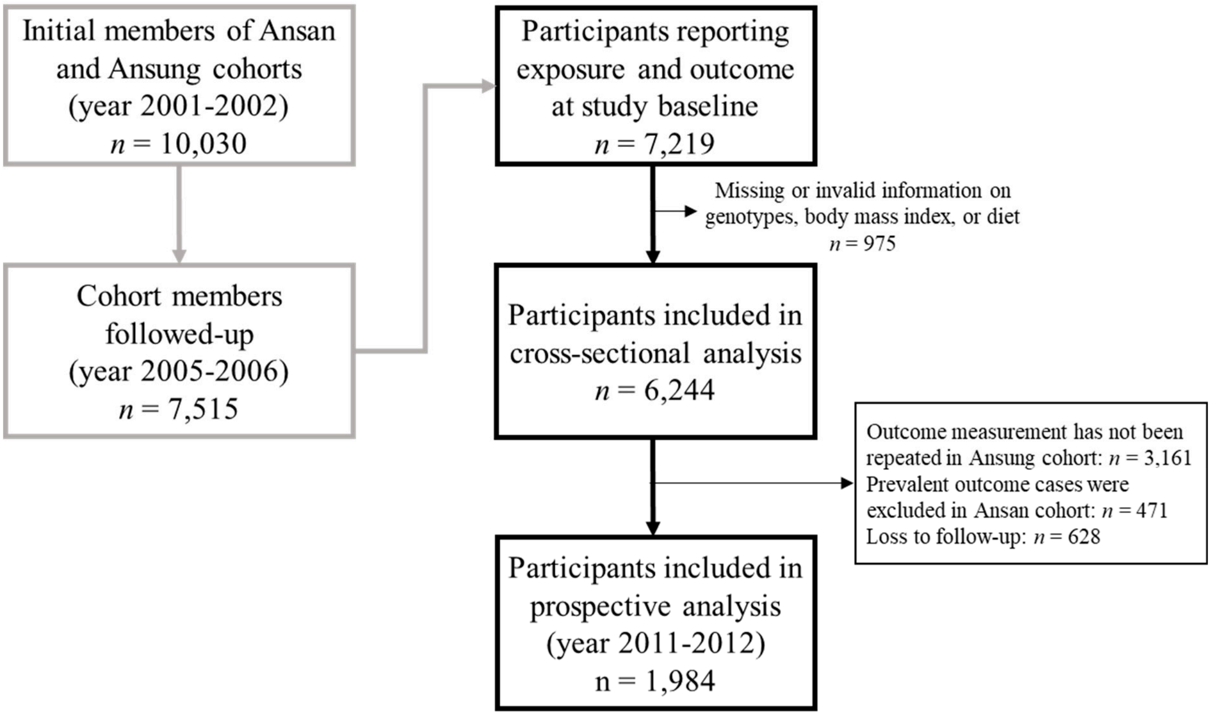

Supplementary Figure S1. Flow diagram for study participants

Supplementary Table S1. Prospective associations between average consumption of the baseline and follow-up assessments and the risk of psychological distress (Psychosocial Wellbeing Index ≥ 27) for 1,984 participants, overall and stratified by *BDNF* Val66Met genotype.

**Beverages**	**Cases/Non-cases**[1] **[Cases/Non-cases]**[2]	**RR (95% CI) for all**		**RR (95% CI) stratified by genotypes**	
		**Model 1**	**Model 2**	**Model 2 for CC**	**Model 2 for CT/TT**
Carbonated soft drinks					
No consumption	147/1,327 [52/398:95/929]	Reference	Reference	Reference	Reference
< 1 cup/week	46/365 [14/122:32/243]	1.23 (0.90, 1.70)	1.26 (0.88, 1.81)	1.14 (0.64, 2.05)	1.45 (0.99, 2.14)
≥ 1 cup/week	14/85 [3/31:11/54]	1.64 (0.98, 2.74)	1.45 (0.95, 2.22)	0.79 (0.24, 2.55)	2.51 (1.38, 4.56)
p for trend		< 0.05	< 0.05	0.72	< 0.01
Coffee					
No consumption	17/162 [8/58:9/104]	Reference	Reference	Reference	Reference
< 1 cup/day	51/426 [15/131:36/295]	1.12 (0.66, 1.88)	1.14 (0.67, 1.93)	0.80 (0.34, 1.87)	1.44 (0.71, 2.92)
1-1.9 cups/day	65/590 [19/184:46/406]	1.12 (0.67, 1.86)	1.15 (0.68, 1.95)	0.71 (0.31, 1.65)	1.54 (0.75, 3.15)
≥ 2 cups/day	74/599 [27/178:47/421]	1.38 (0.83, 2.30)	1.35 (0.78, 2.32)	1.08 (0.49, 2.41)	1.57 (0.74, 3.31)
p for trend		0.12	0.22	0.37	0.40
Green tea					
No consumption	53/336 [21/105:32/231]	Reference	Reference	Reference	Reference
< 4 cups/week	82/708 [21/224:61/484]	0.76 (0.55, 1.06)	0.83 (0.60, 1.16)	0.52 (0.30, 0.93)	0.97 (0.64, 1.47)
4-6 cups/week	44/431 [19/128:25/303]	0.71 (0.48, 1.03)	0.82 (0.56, 1.20)	0.89 (0.50, 1.57)	0.72 (0.45, 1.18)
≥ 7 cup/week	28/302 [8/94:20/208]	0.66 (0.43, 1.03)	0.77 (0.50, 1.19)	0.56 (0.25, 1.22)	0.85 (0.49, 1.45)
p for trend		0.15	0.39	0.65	0.39

Abbreviations: RR, risk ratio; CI, confidence interval. [1]Number of PD cases and non-cases in the overall study population; [2]Number of PD cases and non-cases among CC carriers vs. (indicated as ':') CT/TT carriers. Model 1 was adjusted for age (continuous) and sex. Model 2 was adjusted for age (continuous), sex, marital status (married and living with a spouse or other), monthly income ($\leq 2 \times 10^6$ or $> 2 \times 10^6$ won), educational level (≤ middle school or ≥ high school), occupational type (office worker or other), body mass index (continuous), smoking (pack-years, continuous), alcohol consumption (five categories: non-drinker, former drinker or non-responder, or current drinker: consuming ≤ 15 g/day, 15.1–30 g/day, or > 30 g/day), daily physical activity (four categories: none, < 30 min/day, 30-59 min/day, or ≥ 60 min/day), daily calorie intake (quintiles), prevalent diabetes mellitus and hypertension or physician-diagnosed cardiovascular disease and cancer in the past 2 years (yes or no), *BDNF* Val66Met genotype (CC vs. CT and TT), and consumption of carbonated soft drinks, coffee, and green tea.
